# Multiparametric Regressions and Machine Learning: UAV Imagery for Temperature Prediction of Irrigated Forage Cactus in the Brazilian Semiarid

**DOI:** 10.3390/s26196327

**Published:** 2026-10-07

**Authors:** Eric Gabriel Fernandez Albuquerque da Silva, Thayná Alice Brito Almeida, Raví Emanoel de Melo, João Luis Mendes Pedroso de Lima, José Amilton Santos Júnior, Abelardo Antônio de Assunção Montenegro

**Affiliations:** 1 Department of Agricultural Engineering, Federal Rural University of Pernambuco, Recife 52171-900, Pernambuco, Brazil; eric.fernandez@ufrpe.br (E.G.F.A.d.S.); ravi.melo@ufrpe.br (R.E.d.M.); joseamilton@ufrpe.br (J.A.S.J.); abelardo.montenegro@ufrpe.br (A.A.d.A.M.); 2 Postgraduate Program in Agricultural Engineering, Federal Rural University of Pernambuco, Recife 52171-900, Pernambuco, Brazil; thayna.britoalmeida@ufrpe.br; 3 MARE-Marine and Environmental Sciences Centre, ARNET-Aquatic Research Network, Department of Civil Engineering, Faculty of Sciences and Technology, University of Coimbra, Rua Luís Reis Santos, Pólo II, 3030-788 Coimbra, Portugal

**Keywords:** vegetation indices, crop water status, precision agriculture, artificial intelligence

## Abstract

Vegetation surface temperature is a key indicator of crop water status, particularly in semi-arid regions where water availability constrains agricultural production. This study evaluated whether spectral bands and vegetation indices derived from UAV multispectral imagery can predict the surface temperature of irrigated forage cactus (*Opuntia* spp.) grown with reused water in the Brazilian semiarid region. A randomized block experiment was conducted in Pesqueira, Pernambuco, Brazil. Canopy temperature was measured with a handheld thermal camera (FLIR E6), and multispectral imagery was acquired with a DJI Phantom 4 UAV across three flights. Linear, polynomial, and multiparametric regression models were compared against four machine learning algorithms (Random Forest (RF), epsilon-support vector regression (SVR), Extreme Gradient Boosting (XGBoost), and K-Nearest Neighbors (KNN)), each evaluated over 1500 randomized train/test splits. Vegetation indices were negatively correlated with canopy temperature, consistent with higher vegetative vigor being associated with cooler canopies. The seven-variable multiparametric regression model achieved the best regression performance (validation R^2^ = 64.70%, Nash–Sutcliffe efficiency = 0.84), while KNN was the best-performing machine learning algorithm (mean test R^2^ = 78.11%). The findings suggest that UAV-derived spectral indices represent a promising indirect proxy for estimating forage cactus canopy temperature under the evaluated semiarid conditions. However, independent validation across diverse environments and management conditions is necessary prior to operational implementation as an alternative to direct thermal sensing.

## 1. Introduction

Precision agriculture relies on characterizing the spatial and temporal variability of crop and soil attributes to guide the targeted application of inputs such as fertilizers and irrigation water [1]. Unmanned aerial vehicles (UAVs) have become a central tool in this context, offering spatial and temporal resolution, as well as data acquisition repeatability, that are difficult to achieve with satellite or ground-based platforms [2,3]. When equipped with RGB or multispectral sensors, UAVs allow the derivation of spectral indices capable of capturing environmental and physiological information from reflectance patterns across different wavebands. These indices have been used to identify heterogeneous zones and bare-soil patches [4], to delineate zones of higher agricultural productivity [5], to improve the spatial interpolation of soil moisture and electrical conductivity [6], to estimate leaf area index, chlorophyll and carotenoid content [7], to detect wilting [8] and water stress in crops [9], and to evaluate crop evapotranspiration [10].

Among the physiological attributes accessible through this approach, vegetation surface temperature stands out for its close relationship with plant water status, photosynthetic activity, and overall crop development [11]. Changes in canopy temperature can indicate water-stress conditions and directly affect plant growth and yield, as well as influence pest incidence and development [12,13]. Canopy temperature is physiologically linked to crop water status, as plant water relations affect stomatal regulation, transpiration, and the canopy energy balance. However, canopy temperature alone is insufficient for directly diagnosing plant water stress, with additional physiological or water-balance data required.

This last point is precisely where the literature shows converging but still fragmented evidence. Thermal UAV imagery has been directly used to estimate surface temperature through regression models, with accuracy varying as a function of the temperature range itself. Henn and Peduzzi [14] found larger errors when predicting lower temperatures and smaller errors at higher temperatures, using both drone-mounted and handheld thermal sensors. This dependence on dedicated thermal sensors, however, adds cost and operational complexity, motivating a parallel line of research that instead correlates surface temperature with multispectral vegetation indices, which are cheaper to acquire and already routinely collected for other agronomic purposes. Montes de Oca and Flores [15] showed that individual multispectral indices can be regressed against surface temperature with sufficient accuracy for practical estimation. Similar linear relationships have been reported for land surface temperature in a Pakistani watershed [16] and using NDVI as a single predictor for surface temperature more broadly [17], although the strength of these correlations is not uniform. Using multitemporal imagery, Himayah et al. [18] found that the correlation between spectral indices and surface temperature varied considerably with vegetation cover and phenological state. Taken together, these studies indicate that vegetation indices carry meaningful thermal information, but that the strength and even the functional form (linear vs. non-linear) of this relationship is likely crop- and context-specific, a gap that simple linear regression, the dominant approach in this literature, is not well suited to resolve. Previous studies have established relationships between vegetation indices and canopy temperature using direct thermal measurements [19,20]. Alternatively, some research has investigated multispectral information as an indirect predictor of vegetation temperature [10,15].

Machine learning offers a natural way to address this non-linearity and has already been applied to related environmental prediction problems using temperature-based predictors: Random Forest (RF), Generalized Regression Neural Networks, and Support Vector Regression (SVR) have been used to model reference evapotranspiration [21]; RF, K-Nearest Neighbors (KNN), Decision Trees, Support Vector Regression (SVR), and CatBoost have been compared for predicting temperature under climate-change scenarios [22]; and RF, SVM, and KNN have been used to refine satellite-derived surface temperature estimates [23]. In forage cactus specifically, UAV-derived visible vegetation indices combined with machine learning have already demonstrated potential application for predicting structural and agronomic traits, reinforcing the applicability of data-driven approaches for crop monitoring under semiarid conditions [24]. However, these applications have not yet been systematically extended to the specific problem of estimating crop canopy temperature from UAV-derived multispectral indices, nor have they been directly benchmarked against the multiparametric regression approaches more commonly used in agronomic remote sensing, particularly in semiarid areas.

This gap is particularly relevant for forage cactus (*Opuntia* spp.), a crop of major economic and ecological importance for livestock systems in the Brazilian semiarid region, where water scarcity makes water-status monitoring especially valuable, and where reused water is increasingly adopted for irrigation as a strategy to cope with limited freshwater availability. To date, evaluation of the potential of UAV-derived multispectral indices as an indirect proxy for estimating forage cactus surface temperature has not been properly addressed, nor has the relative performance of classical multiparametric regression versus machine learning algorithms been assessed for this purpose. We hypothesized that (i) vegetation indices more sensitive to chlorophyll content and canopy structure (e.g., GNDVI, NDRE, EVI) would show stronger associations with canopy temperature than raw spectral bands, reflecting the physiological link between vegetative vigor and canopy cooling, and (ii) machine learning algorithms would outperform classical regression models, given the expected non-linearity of this relationship. Accordingly, this study aimed to (1) quantify the correlation between UAV-derived multispectral indices/bands and forage cactus surface temperature, and (2) compare the predictive performance of multiparametric regression models against four machine learning algorithms (RF, SVR, XGBoost, and KNN) for estimating this temperature in forage cactus irrigated with reused water in the Brazilian semiarid region.

## 2. Materials and Methods

### 2.1. Study Area

**Figure 1:**
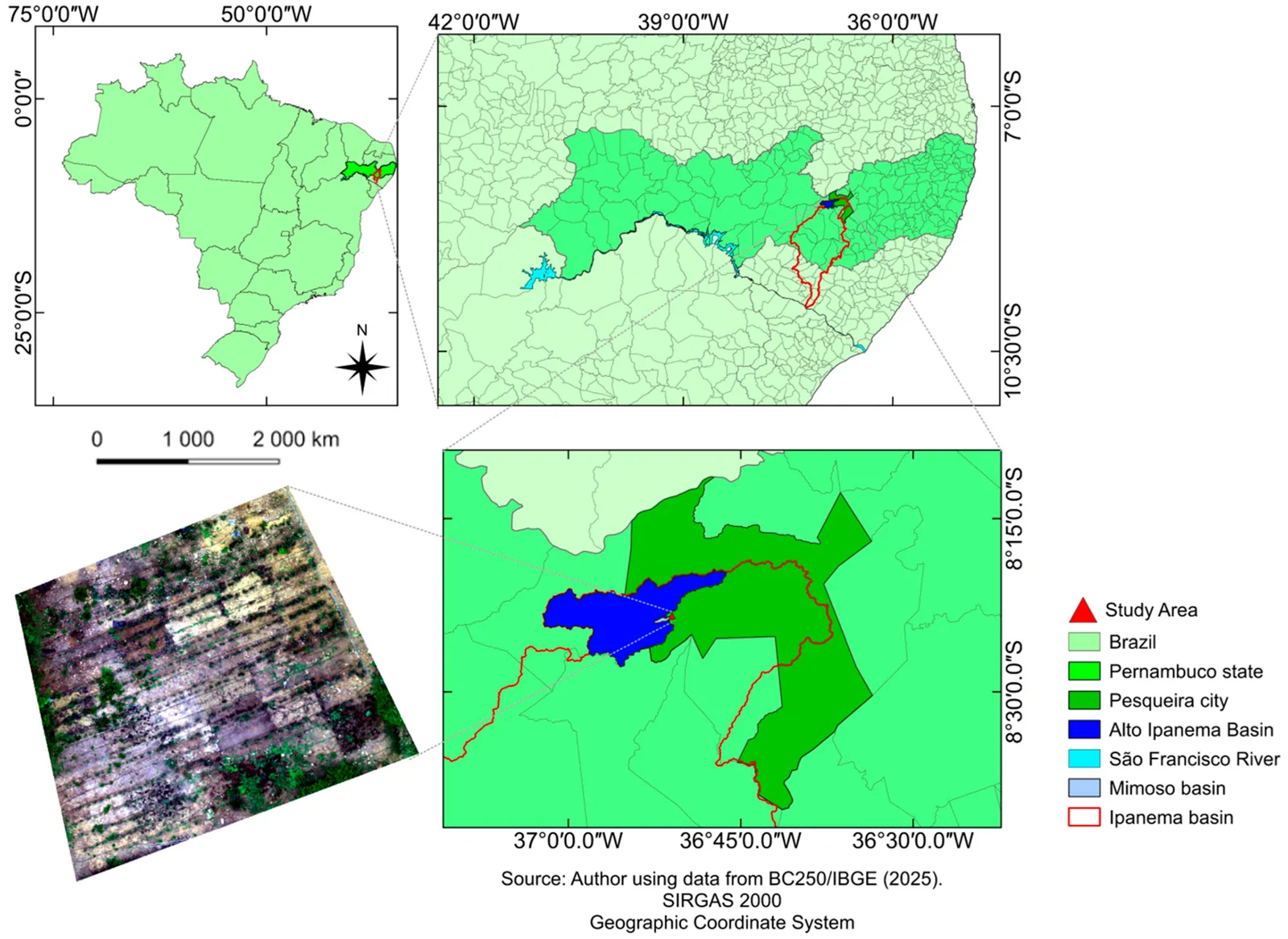
Location of the study area in Pesqueira, Pernambuco state, Brazil, and the experimental site.

The study was conducted at the hydro-agricultural reuse experimental unit located in the Nossa Senhora do Rosário settlement, within the Mimoso Alluvial Valley (MAV), part of the upper Ipanema River basin, itself a tributary of the São Francisco River basin, in the municipality of Pesqueira, Pernambuco (PE), Brazil (Figure 1). The regional climate is classified as Hot Semiarid (BSh) according to the Köppen classification, with a mean annual air temperature of 23 °C and mean annual potential evapotranspiration and precipitation of 2000 mm and 630 mm, respectively [25]. During the experiment, the average temperatures were 25.87 °C, 43.6 °C, and 17.0 °C for the mean, maximum, and minimum temperatures, respectively.

**Figure 2:**
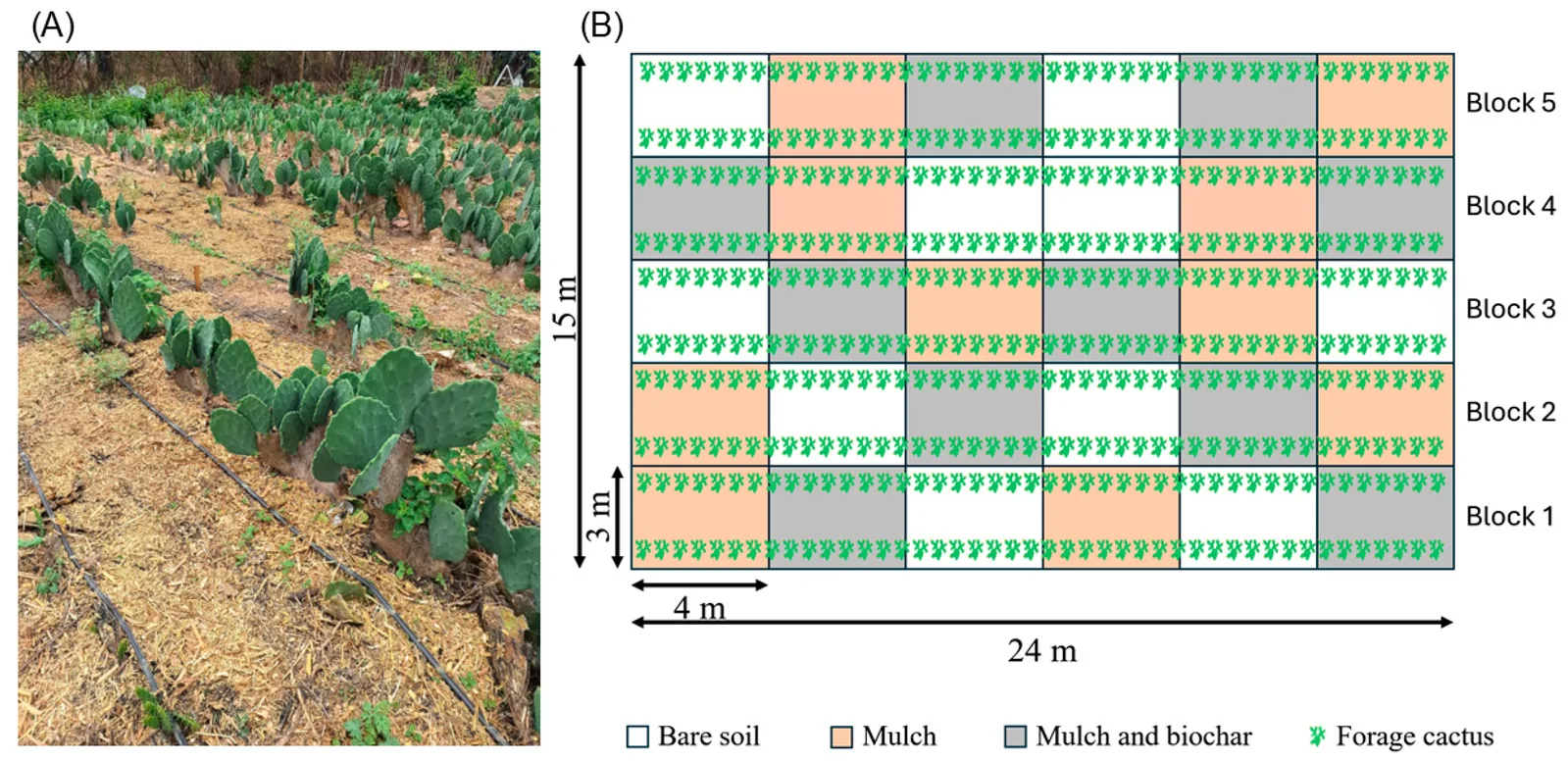
Experimental area showing (**A**) forage cactus with the mulch application and (**B**) layout of the experimental unit under a randomized complete 5-block design (top view), at the hydro-agricultural reuse experimental unit (Nossa Senhora do Rosário—Mimoso Alluvial Valley).

The experiment was arranged in a randomized complete block design (RCBD) with 5 blocks in order to control the effect of terrain slope on the experimental units. Treatments consisted of the application of organic mulch at a rate of 8 Mg ha^−1^ and cashew-nut-shell biochar at a rate of 12 Mg ha^−1^, each replicated 2 times, for a total of 30 experimental plots. Each plot measured 12 m^2^. Irrigation was applied using a localized drip irrigation system supplied with treated domestic wastewater, according to the wastewater reuse methodology described by Mayer et al. [26]. Treatment allocation followed the layout presented in Figure 2.

### 2.2. Thermal Data Acquisition

**Figure 3:**
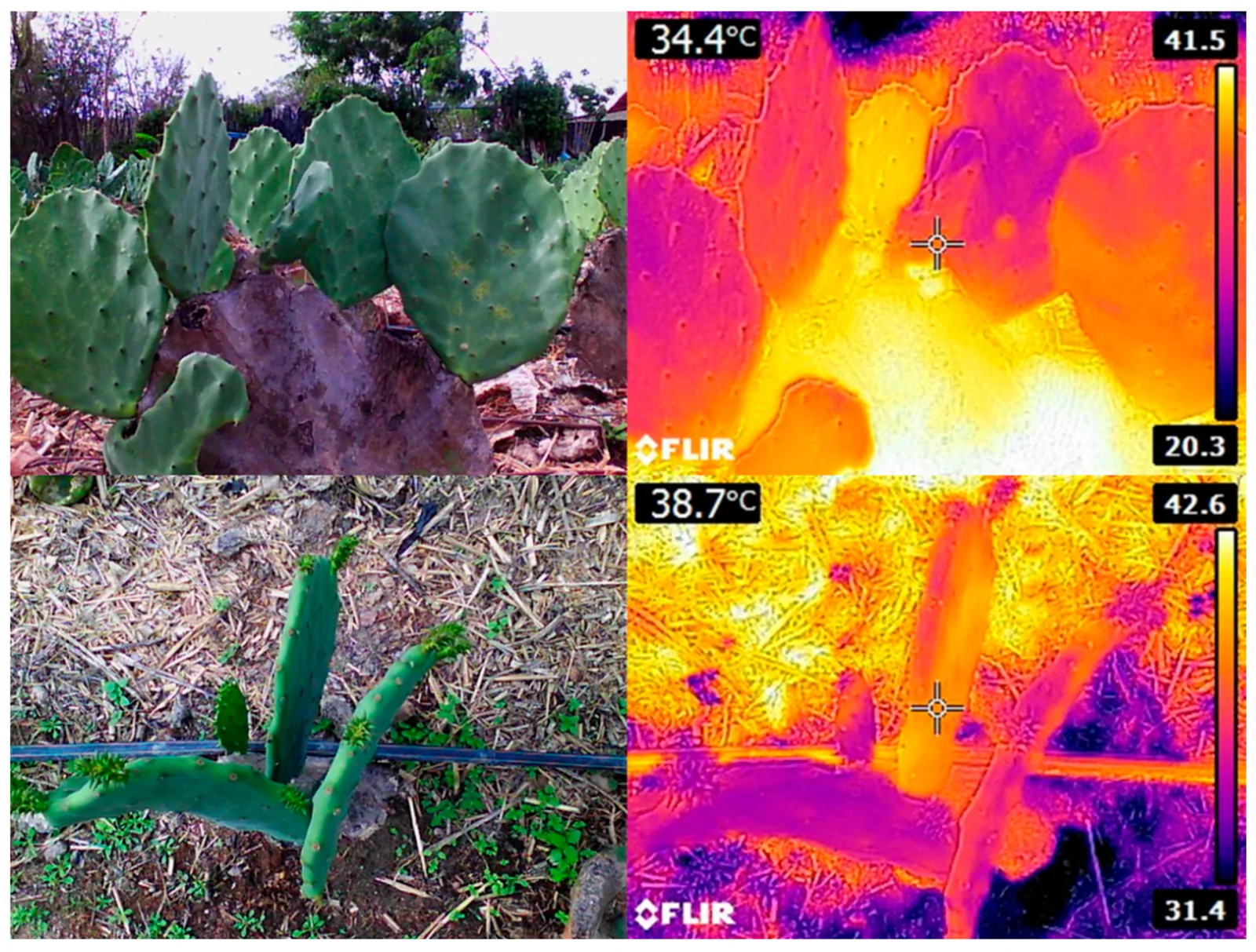
Photographs (**left**) and thermal images (**right**) of forage cactus cladodes for (**top**) frontal view and (**bottom**) top-down view.

Forage cactus canopy (cladode) surface temperature was measured using a handheld thermal infrared camera (FLIR E6, FLIR Systems, Wilsonville, OR, USA), with an optical resolution of 19,200 pixels (160 × 120), a thermal sensitivity of <0.06 °C, and a manufacturer-stated accuracy of ±2%. Thermal images were acquired at two standardized positions relative to the plant: a frontal shot at a fixed distance of 1.0 m and a top-down shot at a fixed distance of 0.5 m from the canopy surface (Figure 3).

Reflected apparent temperature, relative humidity, and object-to-camera distance were set in the camera/software for each acquisition according to on-site environmental conditions recorded at the time of imaging. All thermal images were acquired under clear-sky conditions and within a restricted time window around solar noon to minimize the influence of transient shading and rapidly changing canopy energy balance on the measurements.

Mean cladode surface temperature was extracted from each thermal image using FLIR Thermal Studio software (v. 2.0.69). For each image, a polygon measurement tool was used to delimit the cladode area within the frame, and the mean temperature value within the delimited polygon was recorded as the observation for that sampling point.

### 2.3. Spectral Indices

Multispectral imagery was acquired using a DJI Phantom 4 Multispectral UAV (DJI, Shenzhen, China), equipped with a multispectral camera capturing five discrete spectral bands: blue (450 nm), green (560 nm), red (650 nm), red edge (730 nm), and near-infrared (840 nm). Images were captured at a flight altitude of 30 m, resulting in a ground sampling distance (GSD) of 1.6 cm per pixel. Flight missions were planned with 75% frontal and 75% side image overlap to ensure adequate coverage for photogrammetric reconstruction.

Prior to each flight, a calibrated reflectance panel (CRP) was imaged under the same illumination conditions to correct for ambient light variability and to enable accurate calculation of surface reflectance and, consequently, of the spectral indices derived from it. Raw images were processed in Agisoft Metashape (Agisoft LLC, St. Petersburg, Russia) to generate radiometrically corrected, georeferenced orthomosaics for each spectral band. Spectral indices were subsequently calculated from these orthomosaics in QGIS (v. 3.34.10-Prizren).

Three UAV flights were conducted on 20 January, 25 February, and 25 May 2026. Maximum, average and minimum air temperatures at the time of each flight, monitored using an Onset HOBO RG3 rain gauge/weather logger, were 37.16 °C, 27.71 °C and 21.66 °C for 20 January, 36.72 °C, 25.58 °C and 19.56 °C for 25 February, and 30.86 °C, 22.37 °C and 17.57 °C respectively. Twelve spectral indices and bands were calculated from multispectral imagery, spanning both visible and non-visible wavelengths, as listed in Table 1.

Multispectral and thermal measurements were obtained during the same field campaigns. For each campaign, one plant was selected from each experimental plot, resulting in 30 paired observations per acquisition date. Multispectral imagery was acquired during the UAV flight, and the corresponding thermal image was captured immediately after the flight.

### 2.4. Statistical Analysis

Descriptive statistics (mean, minimum, maximum, standard deviation, and coefficient of variation) were calculated for all measured variables, based on a total of 90 observations per variable. The experimental dataset comprised 30 experimental plots evaluated across three acquisition dates (20 January, 25 February, and 25 May 2026). For each sampling event, one plant was selected from each plot, and the corresponding canopy temperature and spectral information were obtained, resulting in 30 paired observations per date and 90 observations in total. Analysis of variance (ANOVA) was performed to test for the effect of soil-management treatment on forage cactus canopy surface temperature, separately for each sampling date, and to evaluate whether the randomized block design was warranted given the observed spatial variability.

The coefficient of variation (CV) was classified according to the criteria proposed by Warrick and Nielsen [36], in which CV values below 12% indicate low variability, values between 12% and 60% indicate moderate variability, and values above 60% indicate high variability. Because the studied variables differed substantially in scale and units, min–max normalization was applied to the predictors and response variable within the training partition for the classical regressions. The minimum and maximum values were calculated exclusively from the training data and subsequently used to rescale both the training and test data to the 0–1 range.

### 2.5. Regression Models

The data were fitted using linear regression, second- and third-degree polynomial regression, and multiparametric (2 to 7 factors), each tested against the sets of indices and bands extracted from the images. To determine the regression equations and their respective coefficients of determination, an algorithm was developed in Python (v.3.13.7) using the NumPy library (v.2.4.2) for matrix operations.

Regression equations were estimated using the ordinary least squares method to minimize residual errors. Applying this method to a simple linear equation (Equation (1)):

*y* = *a*_0_ − *a*_1_ × *x* − *e* (1)

where e is the associated residual error.

By applying the method of least squares and considering the sum of squares to determine the sum of the residuals (Equation (2)), the derivative with respect to each coefficient is calculated (Equation (3)).

(2) $$\mathrm{Sr}=\sum_{i=1}^{n}{\left(y_{\mathrm{predicted}}-y_{\mathrm{observed}}\right)}^{2}=\sum_{i=1}^{n}{\left(y_{i}-a_{0}-a_{1}\times x\right)}^{2}$$

(3) $$\frac{\partial \mathrm{Sr}}{\partial a_{0}}\;\mathrm{and}\;\frac{\partial \mathrm{Sr}}{\partial a_{1}}$$

These equations can be rearranged into matrix form; inverting matrix *A* then yields the model coefficients (matrix Z) (Equation (4)):

[*Z*] = [*A*]^−1^ × [*B*] (4)

Once the regression coefficients were obtained, the estimated values of the dependent variable were calculated by substituting the coefficients into the fitted equation (Equation (5)). Model performance was then assessed based on the explained variability (Equation (6)) and total variability (Equation (7)), which were used to calculate the coefficient of determination (R^2^) (Equation (8)) and the adjusted coefficient of determination (R^2^a). Additionally, the Nash–Sutcliffe efficiency (NSE) (Equation (10)) and percent bias (PBIAS) (Equation (11)) were calculated to further assess model performance and agreement between observed and estimated values.

(5) $$\hat{y}=a_{0}+a_{1}\times x$$

(6) $$\mathrm{VE}=\sum_{i=1}^{n}{\left(\hat{y}-\overset{-}{y}\right)}^{2}$$

(7) $$\mathrm{VT}=\sum_{i=1}^{n}{\left(y-\overset{-}{y}\right)}^{2}$$

(8) $$R^{2}=\frac{\mathrm{VE}}{\mathrm{VT}}$$

(9) $$R^{2}a=1-\left(1-R^{2}\right)\times \frac{n-1}{n-p-1}$$

(10) $$\mathrm{NSE}=1-\frac{\sum_{i=1}^{n}{\left(y-\hat{y}\right)}^{2}}{\sum_{i=1}^{n}{\left(y-\overset{-}{y}\right)}^{2}}$$

(11) $$\mathrm{PBIAS}=100\times \frac{\sum_{i=1}^{n}\left(y-\hat{y}\right)}{\sum_{i=1}^{n}y}$$

where $\hat{y}$ is the estimated value of the dependent variable, $\overset{-}{y}$ is the mean of the dependent variable, and y is the observed value of the dependent variable.

The same methodological procedure was applied to all regression models implemented in the algorithm. For each regression type, the algorithm systematically evaluated all possible combinations of predictor variables within each predefined number of factors and identified the best-performing model based on the highest coefficient of determination. The full source code is available on GitHub [https://github.com/Fernandez-E/Regressao, accessed on 27 March 2026]. All models were calibrated and validated using 75% and 25% of the data, respectively.

### 2.6. Machine Learning Models

In addition to the regression approaches, four supervised machine learning regression algorithms were applied to estimate forage cactus canopy temperature: RF, SVR, XGBoost, and KNN, all implemented using the Scikit-learn library (v. 1.9.0). All code was developed and executed in a Google Colab notebook using Python 3.

The training and test sets were randomized, totaling 1500 runs per algorithm. A search for optimal hyperparameters was also conducted for each algorithm: “c” for SVR, “n_estimators” for RF and XGB, and “n_neighbors” for KNN. These hyperparameter ranges align with observations made by Arias-Rodriguez et al. [37], who used RF and SVM for water quality monitoring; Kai et al. [38], who used deep learning with KNN for sugarcane classification; and Hirooka et al. [39], who predicted cowpea growth using KNN, SVM, and RF. All other hyperparameters were kept at the models’ default settings. The hyperparameters used in the search procedure are presented in Table 2.

After tuning, the parameters used are presented in Table 3.

Machine learning models were evaluated using the Mean Absolute Error (MAE), Root Mean Squared Error (RMSE), and coefficient of determination (R^2^) metrics. The coefficient of determination from each run was recorded to characterize the frequency distribution and assess the stability of the resulting model.

Classical regression models were initially calibrated and validated using a single 75%/25% partition of the dataset, with 75% of the observations used for calibration and the remaining 25% for validation. To address the difference in validation procedures between the modelling approaches, the machine learning models were evaluated separately using 1500 randomized train/test partitions. Therefore, the performance estimates from the classical regression and machine learning approaches were interpreted according to their respective validation procedures and were not treated as strictly equivalent performance estimates.

**Figure 4:**
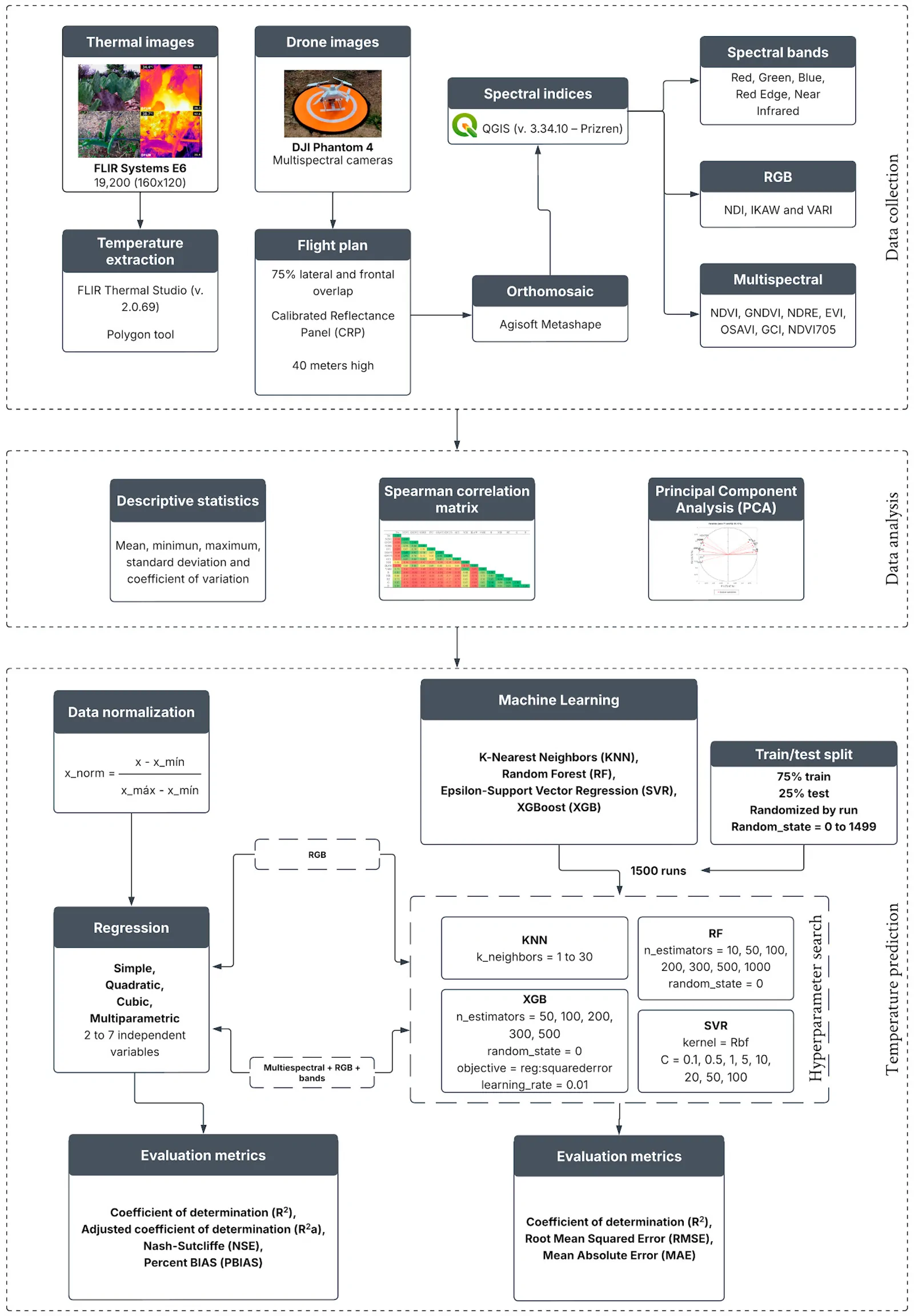
Overview of the workflow used to predict forage cactus canopy temperature from UAV-derived spectral information.

The methodology employed in this study is summarized in the flowchart shown in Figure 4.

## 3. Results

In this study, we collected data on various measured variables during the fieldwork phase, which comprised a total of 90 observations for each variable. This research was conducted at the hydro-agricultural reuse experimental unit situated in the Nossa Senhora do Rosário settlement. The descriptive statistics summarizing these observations are provided in Table 4, offering insight into the characteristics and distributions of the variables analyzed.

Plant temperature (Tm) averaged 39.37 °C across the evaluated period and conditions, ranging from 31.85 to 46.15 °C (Table 3). The coefficient of variation (9.6%) indicates low data variability, suggesting low variability according to the classification by Warrick and Nielsen [36].

Among the vegetation indices, NDVI showed a mean of 0.596 and a range from 0.287 to 0.840. The CV of 21.2% indicates moderate variability. A similar pattern was observed for OSAVI, which showed a mean of 0.596 and a CV of 21.2%. GNDVI showed a mean of 0.457 and high dispersion (CV = 43.8%) compared to NDVI. NDRE showed a mean of 0.134 and a CV of 60.3%, classified as high variability. EVI showed a mean of 1.343 and a CV of 156%, indicating substantial dispersion among observations. The NDVI_705_ index showed a mean of 0.511 and moderate variability (CV = 20.9%), whereas GCI showed considerably dispersed values—mean of 2.650, a maximum of 10.476, and CV of 78.6%—indicating high sensitivity to chlorophyll variations [40] and plant nutritional status [41].

Regarding RGB indices, NDI, IKAW, and VARI showed means of 0.189, −0.052, and 0.40, respectively. NDI showed moderate variability (CV = 52.3%), whereas IKAW exhibited a CV exceeding 300% and VARI showed 69.2%, highlighting high variability for all three indices. The near-infrared (NIR) band showed the highest mean values (22,488), followed by the RE (18,171), R (6337), G (10,391), and B (3094) bands. The NIR and RE bands exhibited moderate coefficients of variation (40.3% and 53.0%, respectively). In contrast, the visible bands showed greater dispersion, especially the blue band (CV = 94.7%).

**Figure 5:**
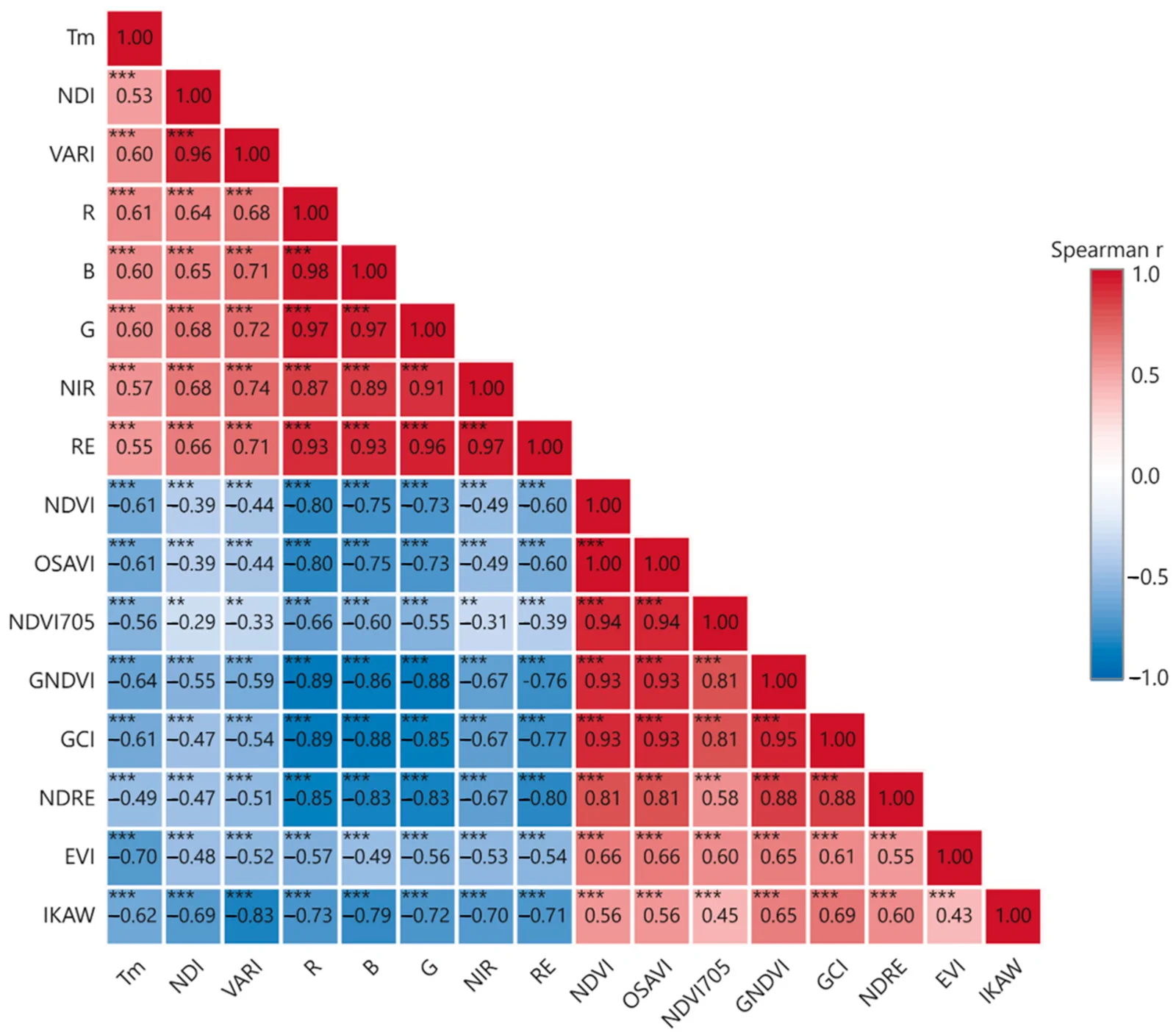
Spearman correlation matrix for the temperature and indices. ** q < 0.01, *** q < 0.001.

Plant temperature showed a negative linear correlation (Figure 5) with most of the calculated vegetation indices—with the exception of NDI and VARI—reinforcing the finding that plants with greater vegetative vigor tend to exhibit lower leaf temperatures. This finding is consistent with Tamboto et al. [42], who found negative correlations between pine tree temperature and the NDVI index in Indonesia using satellite imagery, thereby demonstrating the potential to identify stressed crops via multispectral imaging.

The strongest associations were observed for GNDVI (r = −0.80), EVI (r = −0.80), IKAW (r = −0.79), and VARI (r = 0.75), suggesting that these indices are more sensitive to temperature-related physiological changes; notably, NDVI is sensitive to changes in vegetation cover, while GNDVI is sensitive to changes in chlorophyll content [8,27,28,43]. Nandy and Kumar [44] similarly note that rising temperature leads to a reduction in vegetative vigor and, consequently, a decrease in NDVI. Singh et al. [17] noted that NDVI can serve as a predictor of temperature for various surface types through regression models.

The stronger negative correlations observed between GNDVI and EVI and canopy temperature, compared to the red-edge indices NDRE and NDVI705, likely reflect differences in the spectral and structural properties captured by these indices. GNDVI is particularly sensitive to chlorophyll-related variations in vegetation, while EVI incorporates sensitivity to canopy structure and reduces soil-background and atmospheric effects. In contrast, red-edge indices are especially advantageous under dense and high-biomass canopies due to their lower susceptibility to saturation. Therefore, a plausible explanation for the comparatively weaker relationships of NDRE and NDVI705 in the present study is that the forage cactus canopy may not have reached the density or biomass range where red-edge sensitivity offers a distinct advantage. Alternatively, canopy-temperature variability under the evaluated conditions may have been more closely associated with chlorophyll-related spectral responses and canopy architecture captured by GNDVI and EVI. However, these mechanisms should be regarded as plausible interpretations rather than confirmed physiological responses, since chlorophyll content, biomass, and detailed canopy structural traits were not directly measured in this study.

Although GNDVI, EVI, IKAW, and VARI exhibit significant correlations with canopy temperature, these results do not constitute direct evidence that these indices can diagnose plant water stress. Canopy temperature is physiologically linked to transpiration and stomatal regulation, as reduced stomatal conductance limits evaporative cooling and increases canopy temperature. However, canopy temperature is also influenced by environmental variables, including air temperature, radiation, relative humidity, vapor pressure deficit, and wind speed [45,46]. Consequently, the present findings confirm an association between UAV-derived spectral indices and canopy temperature, but not between spectral indices and plant water-stress status. Establishing the latter would require independent validation using physiological or water-balance indicators, such as plant water potential, stomatal conductance, soil water content, CWSI, or evapotranspiration, which is beyond the scope of this study.

**Figure 6:**
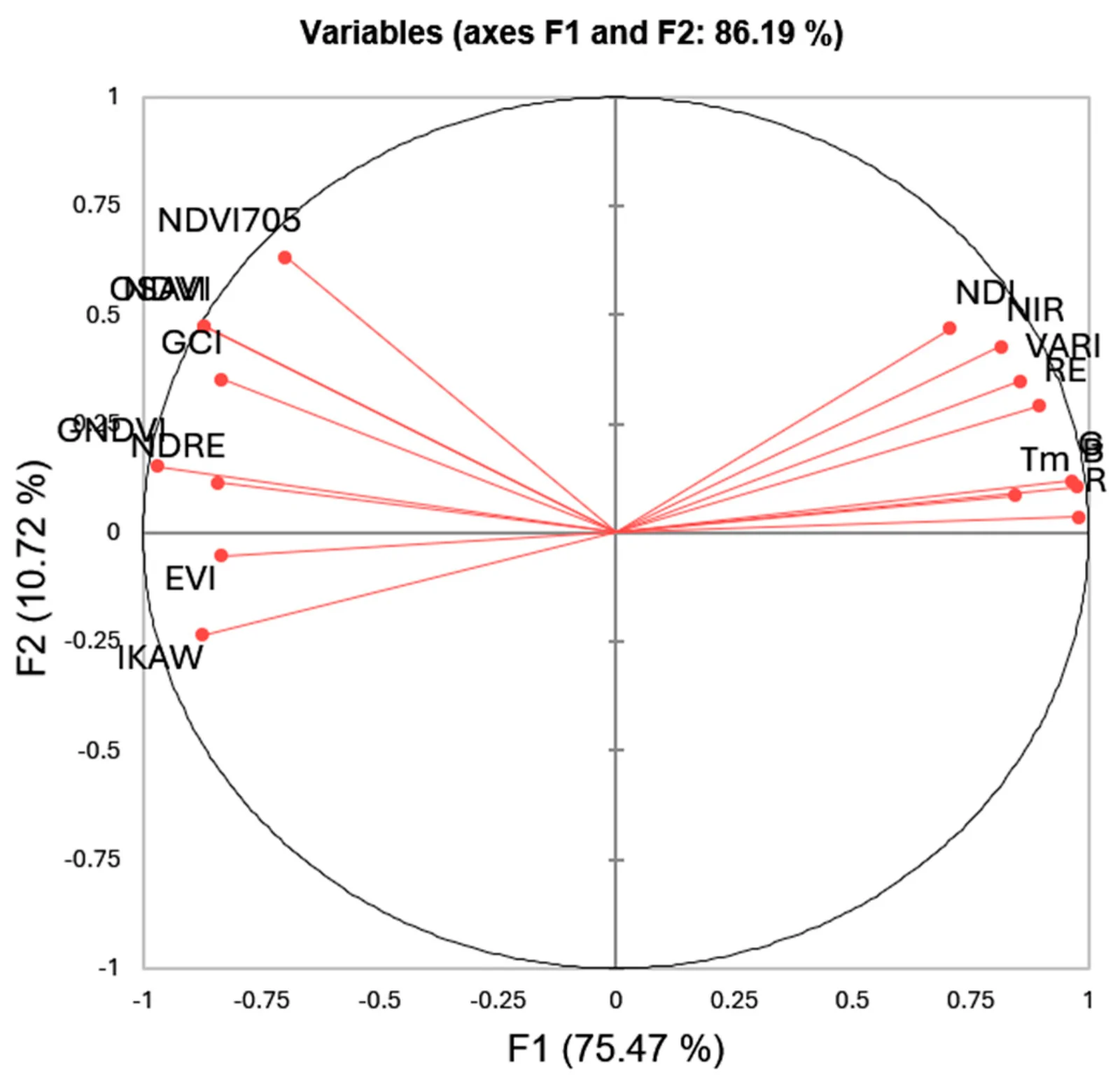
Principal Component Analysis (PCA) correlation circle of canopy temperature (Tm), spectral bands, and vegetation indices.

The PCA results (Figure 6) reinforce the correlation patterns, revealing two major groups: one characterized by positive correlations among spectral bands and the VARI and NDI indices, and the other by negative correlations involving multispectral indices and the IKAW. The first two principal components (F1 = 75.47% and F2 = 10.72%) explain 86.19% of the total variance. The F2 component played a role in the secondary separation of index sensitivity.

Red-edge-based indices, such as NDVI705 and NDRE, were positioned in the upper-left quadrant, showing a slight separation from indices like EVI (lower-left quadrant). This divergence captures structural nuances of the canopy, as NDRE and NDVI705 mitigate the early saturation effect observed with traditional NDVI under high biomass density, whereas EVI incorporates corrections for atmospheric effects. This differential sensitivity underscores the importance of a multivariate approach for the precise characterization of crop biophysical behavior using sensors.

To evaluate whether soil management influenced forage cactus canopy temperature, an analysis of variance (ANOVA) was performed separately for each sampling date. The results are summarized in Table 5.

It is noteworthy that there was no significant difference among treatments regarding the surface temperature of the forage cactus on any of the data collection dates; although mulch is frequently associated with reduced soil temperature and moisture conservation [47], factors that indirectly influence crop temperature due to reduced water stress, no significant differences in plant surface temperature were observed among the treatments.

**Figure 7:**
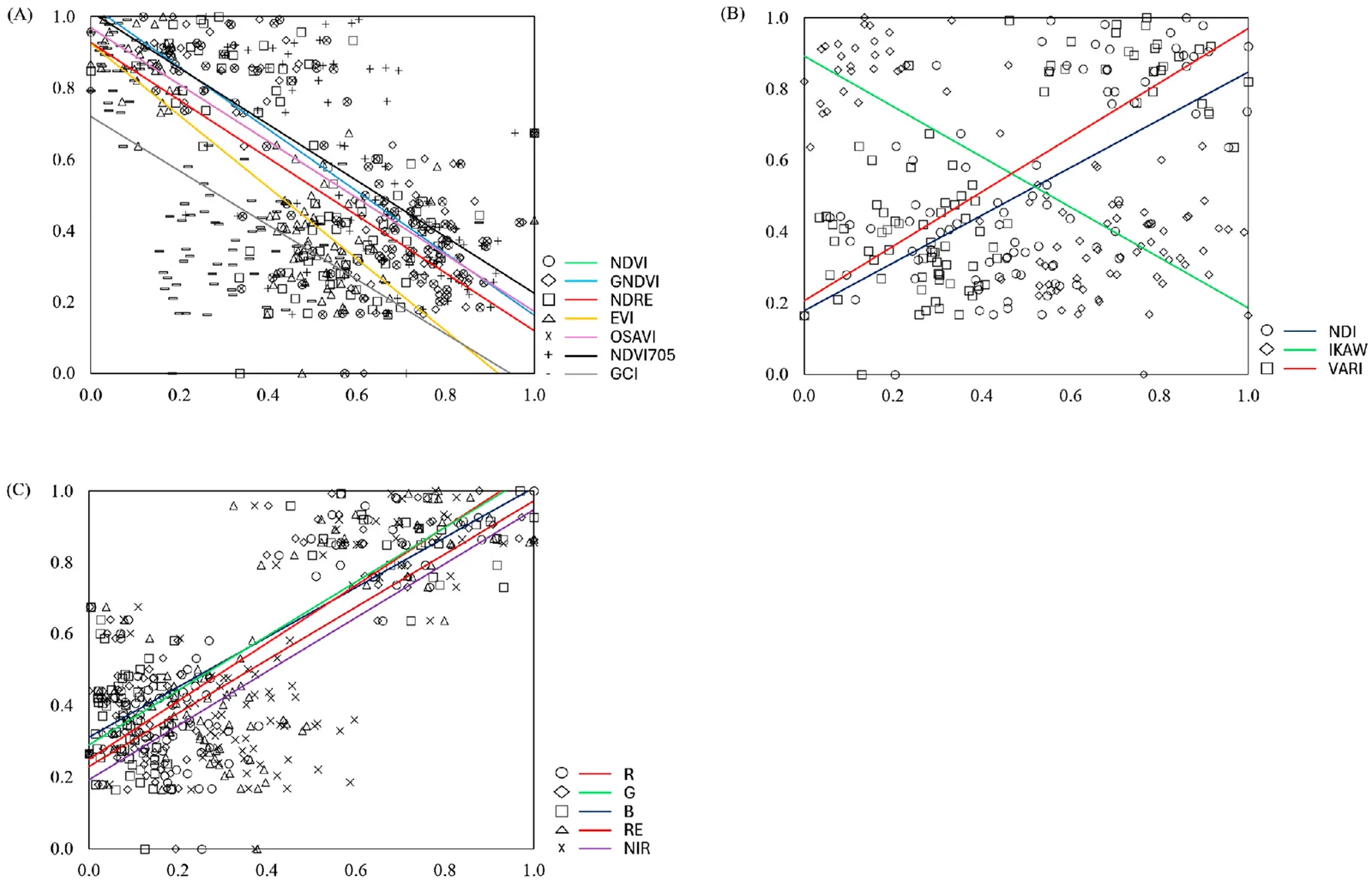
Relationship between temperature and (**A**) multispectral indices, (**B**) RGB indices, and (**C**) spectral bands.

To further investigate the relationships between canopy temperature and the spectral variables, simple linear regressions were performed using the normalized dataset. The resulting relationships are presented in Figure 7.

It is noteworthy that all multispectral indices decrease as temperature rises—as does the RGB IKAW index—whereas all spectral bands and the RGB NDI and VARI indices show an increasing trend with the increase in forage cactus temperature. To quantify the predictive potential of these variables, a series of regression models with increasing complexity were developed. The calibration and validation performances of the models are summarized in Table 6.

The increase in the coefficients of determination (R^2^) and Nash–Sutcliffe values as more variables are added to the model demonstrates a gain in information regarding temperature through the inclusion of spectral indices. Although the calibrated models explain the data well, performance declines during validation, yet the values remain satisfactory. Nandy and Kumar [44] observed surface temperature estimates using NDVI and a linear model achieving R^2^ values of 82% to 88%, indicating that the index is a good predictor of the variable.

To evaluate whether satisfactory predictions could also be achieved using only RGB-derived information, additional regression models were developed using the RGB indices (NDI, IKAW, and VARI). The resulting equations and performance metrics are presented in Table 7.

Compared with the multispectral models, the RGB-based regressions exhibited consistently lower predictive performance. The best RGB model (cubic regression) achieved a validation R^2^ of 55.70%, whereas the best multiparametric RGB model reached only 44.70%, both substantially lower than the 64.70% obtained with the multispectral MP7 model. These findings indicate that although RGB indices capture part of the temperature variability, the inclusion of red-edge and near-infrared information considerably enhances model accuracy by providing greater sensitivity to canopy physiological conditions.

**Figure 8:**
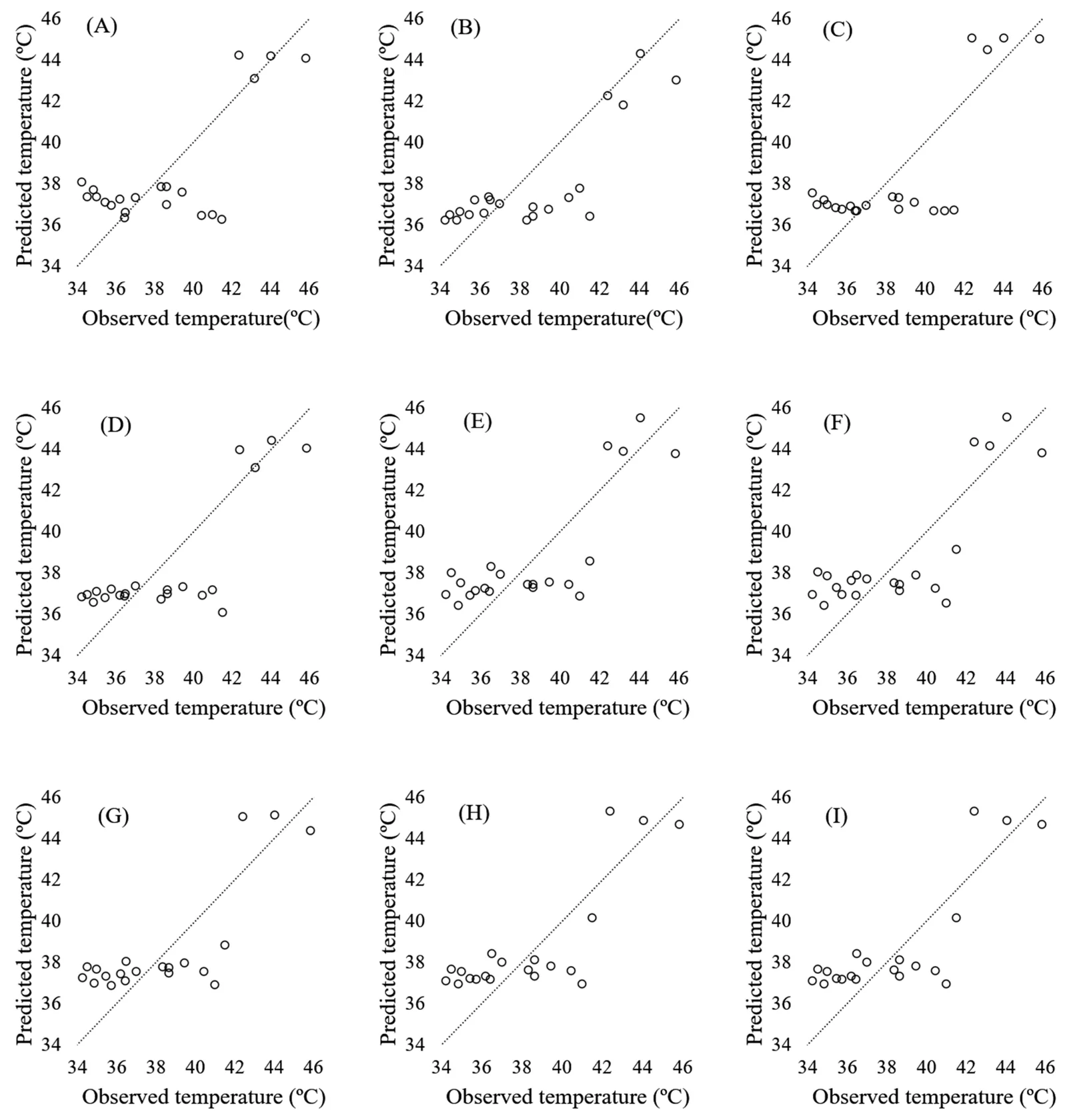
Validation of the regression models using 1:1 scatter plots between observed and predicted forage cactus canopy temperatures for (**A**) simple, (**B**) quadratic, (**C**) cubic, (**D**) MP2, (**E**) MP3, (**F**) MP4, (**G**) MP5, (**H**) MP6 and (**I**) MP7 regressions.

Following model calibration and validation, the agreement between observed and predicted canopy temperatures was assessed using 1:1 scatter plots (Figure 8).

It can be observed that, in general, the lower temperatures recorded (up to 37 °C) show a slight tendency toward overestimation, whereas temperatures up to 42 °C show a tendency toward underestimation. The dispersion observed at lower temperatures aligns with findings by Henn and Peduzzi [14], who, using a drone equipped with a thermal sensor and a handheld thermal camera, achieved better predictions for higher temperatures; a similar pattern was observed with predictions based on spectral indices.

**Figure 9:**
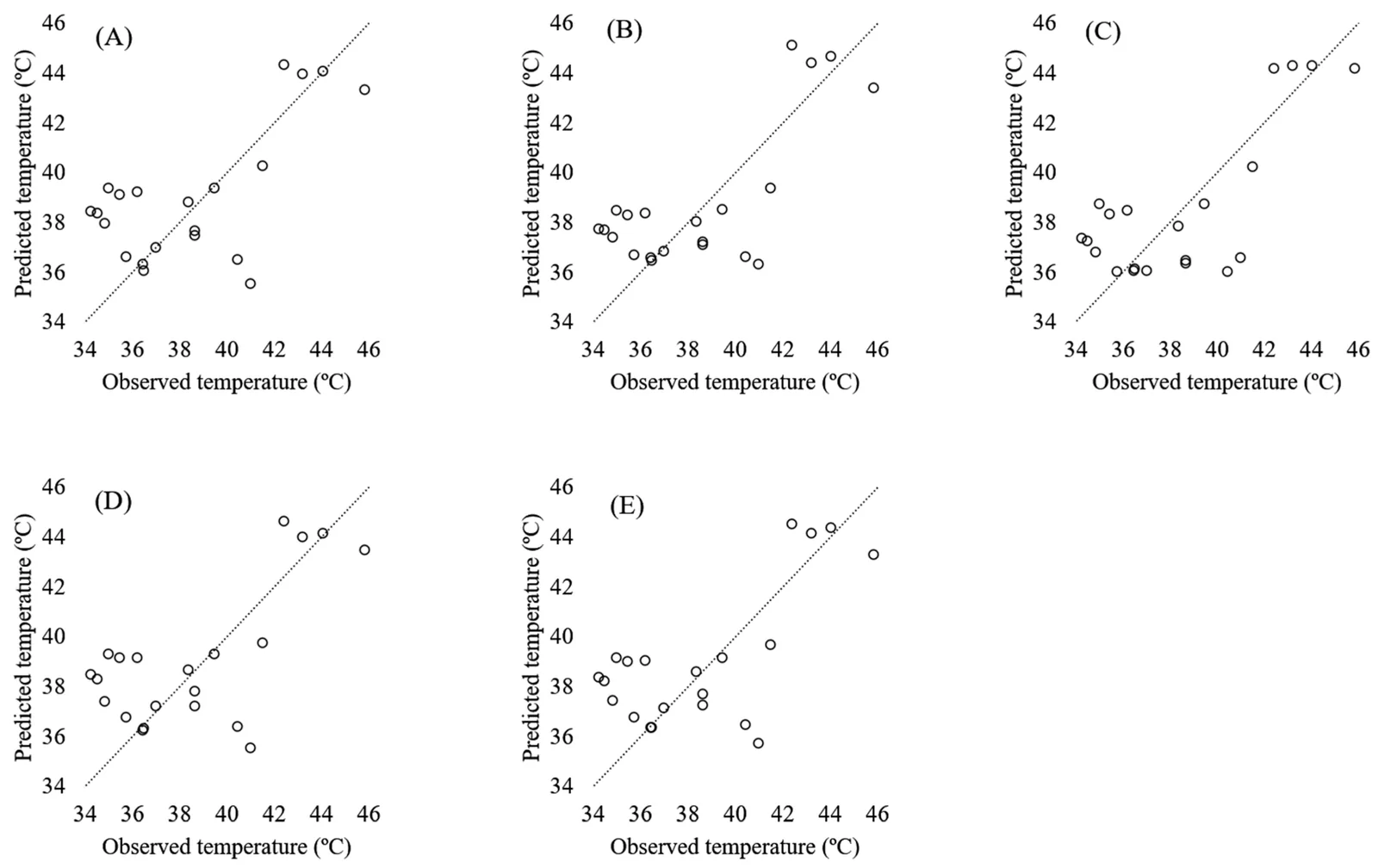
The 1:1 scatter plots for validation of the regression models for RGB indices for (**A**) simple, (**B**) quadratic, (**C**) cubic, (**D**) MP2, (**E**) MP3 regressions.

Thus, based on the analyses conducted, the 7-factor linear model stands out as the best model for estimating forage cactus temperature. For the RGB indices, the scatter plots are shown in Figure 9.

The results obtained and the performance metrics for the machine learning models after 1500 runs are presented in Table 8.

Among the evaluated algorithms, for all bands and indices, KNN demonstrated the best predictive performance, with the lowest MAE (1.3187) and RMSE (1.6996) and the highest coefficient of determination (R^2^ = 78.11%). The Random Forest and SVR models showed intermediate performance, with R^2^ values of 75.83% and 75.61%, respectively.

The predictive performance observed in this study (R^2^ = 0.6212–0.7811; RMSE = 1.6996–2.2392 °C) was lower than that reported by Teshome et al. [10], who achieved R^2^ values above 0.90 and RMSE values between 0.95 and 1.77 °C, depending on crop and algorithm. Although RF was the best-performing model in their study, KNN achieved the highest performance in the present dataset, even when only RGB-derived information was used. These contrasting results suggest that model performance is highly dependent on the dataset and application, and may be affected by crop characteristics, predictor composition, environmental variability, and experimental conditions.

The predictive performance observed in this study aligns with the increasing application of UAV spectral data for estimating crop thermal responses. Montes de Oca and Flores [15] estimated vegetation temperature using UAV multispectral indices and found that the Green Chlorophyll Index exhibited the strongest correlation with reference temperature (R^2^ = 0.71), demonstrating the capacity of non-thermal spectral data to explain variability in vegetation temperature. Similarly, Teshome et al. [10] combined UAV-derived vegetation indices, ground canopy temperature, and machine learning to estimate crop thermal and evapotranspiration responses. Additional studies integrating multispectral and thermal UAV observations have shown that spectral and thermal data provide complementary insights for crop water-status assessment [48,49,50]. In contrast to research validating thermal responses against irrigation treatments, stomatal conductance, soil moisture, or CWSI, the present study focuses on the indirect estimation of canopy temperature and does not directly assess plant water stress.

The predictive performance obtained in the present study is consistent with previous applications of UAV-based vegetation indices and machine learning to forage cactus. Silva et al. [5], using RGB-derived vegetation indices and Random Forest, successfully predicted structural and agronomic traits of forage cactus under different agricultural systems. These findings reinforce the capacity of UAV-derived spectral information combined with machine learning to support data-driven estimation of forage cactus attributes, although model performance may depend on the target variable, sensor configuration, and experimental conditions.

**Figure 10:**
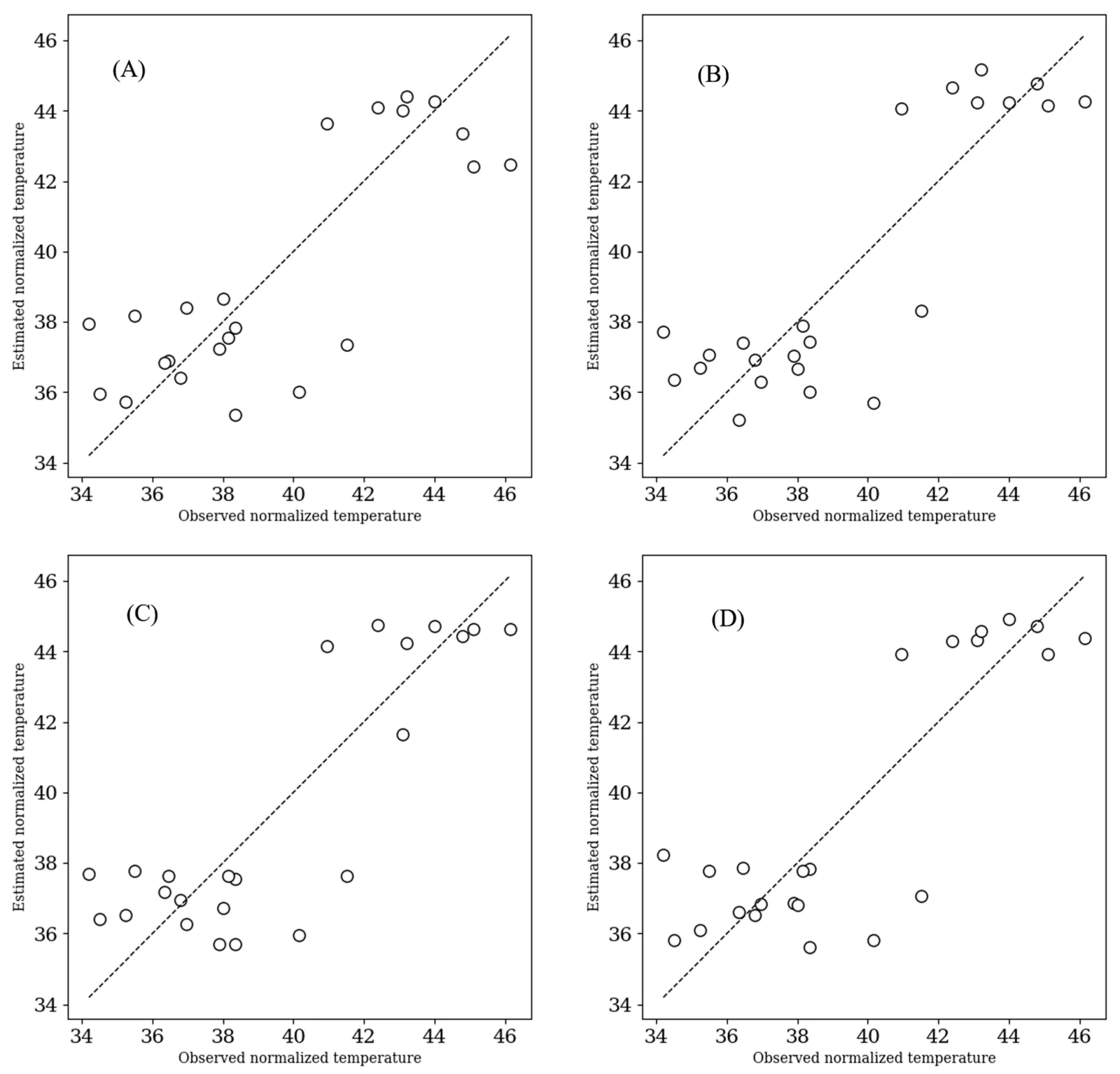
The 1:1 scatter plots for (**A**) KNN, (**B**) RF, (**C**) XGBoost and (**D**) SVM.

The scatter plots for the models with all indices and bands and random_state = 0 for train/test split are presented in Figure 10.

**Figure 11:**
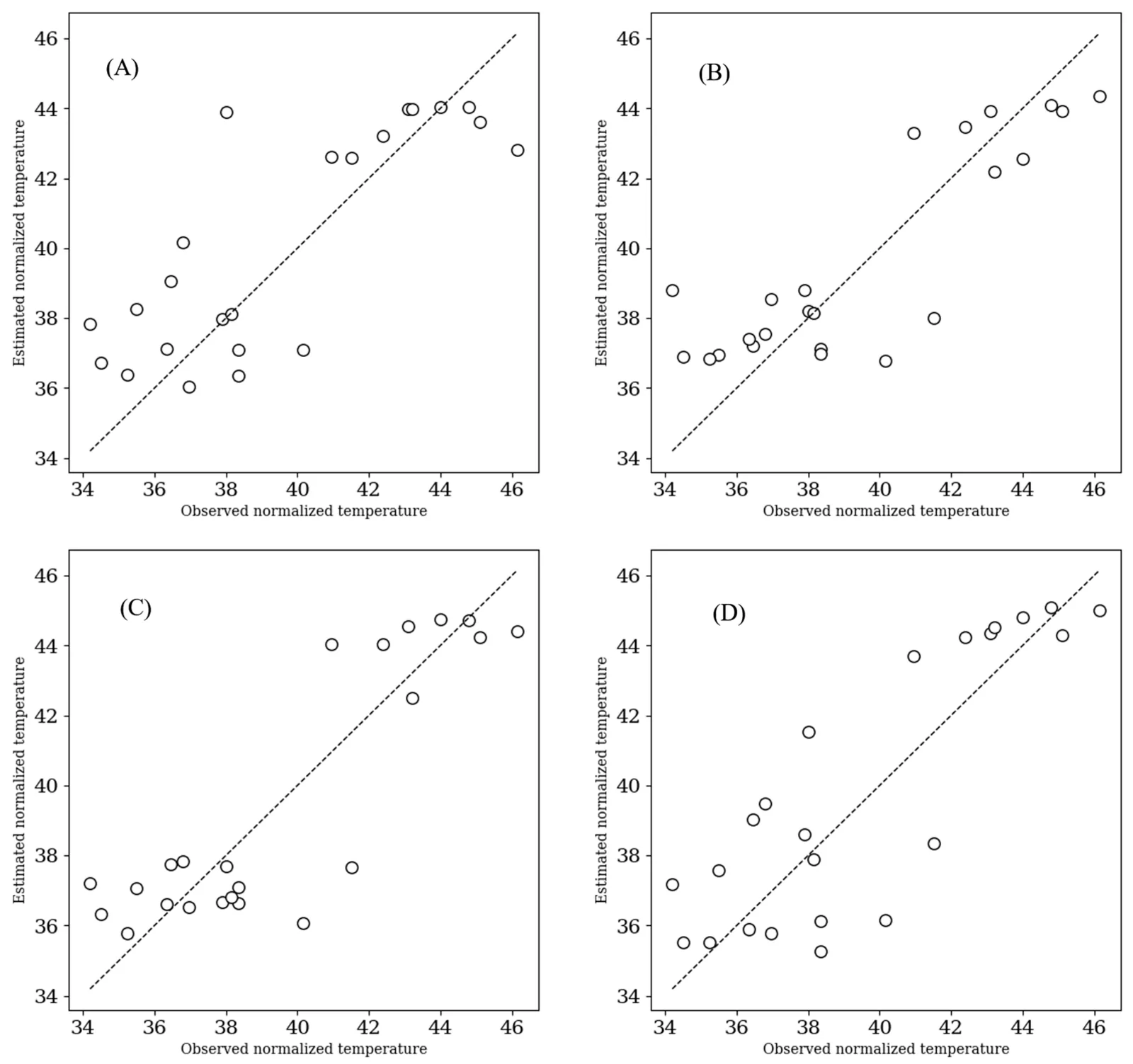
The 1:1 scatter plots for (**A**) KNN, (**B**) RF, (**C**) XGBoost and (**D**) SVM for RGB indices.

For the RGB indices, the 1:1 scatter plots are presented in Figure 11.

**Figure 12:**
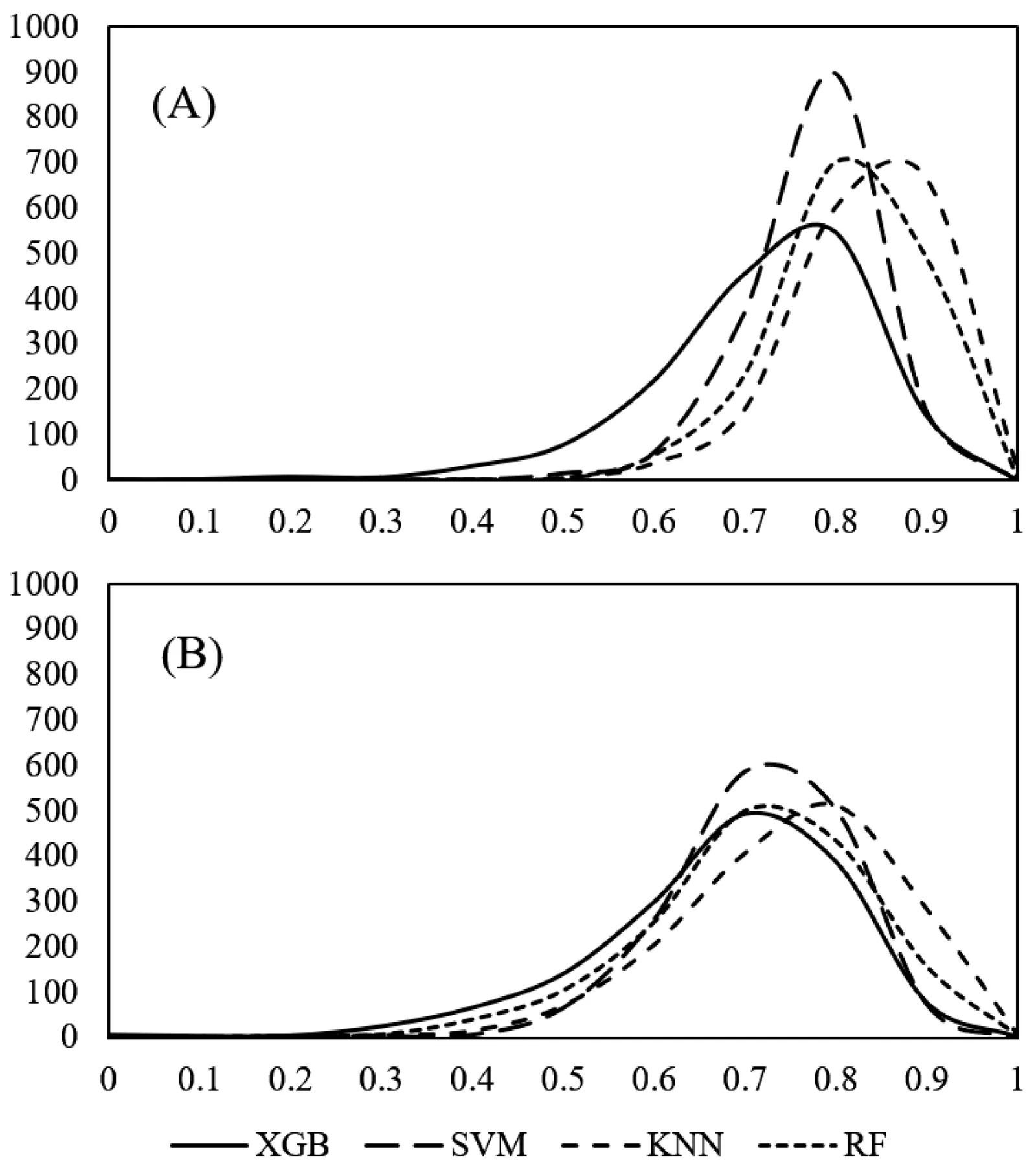
Frequency distribution of coefficients of determination for machine learning models for (**A**) all indices and (**B**) RGB indices.

The frequency distribution for the evaluated models is presented in Figure 12.

Models show greater dispersion when using only RGB indices, whereas the use of multispectral indices, RGB, and spectral bands yields greater consistency across runs—indicating better temperature estimation—even though RGB-based indices employing machine learning do exhibit a good coefficient of determination.

The remote-sensing literature emphasizes the importance of assessing model robustness under diverse scene and acquisition conditions, as variations in illumination, atmospheric conditions, sensor characteristics, and scene composition can influence the transferability of learned representations [51]. This consideration is especially pertinent to the current study, given that the three UAV campaigns were conducted under different environmental conditions.

Therefore, the present results should be interpreted specifically as evidence of the potential of UAV-derived spectral information for indirect canopy temperature estimation, rather than as evidence of plant water-stress diagnosis, which would require independent physiological or soil-water measurements.

## 4. Discussion

Canopy temperature reflects the combined effects of plant physiological regulation and energy exchange with the surrounding atmosphere. When water is sufficiently available, stomatal opening and transpiration facilitate evaporative cooling. In contrast, limited water supply reduces stomatal conductance and transpiration, which decreases latent heat dissipation and raises canopy temperature. As a result, canopy temperature is widely used as a plant-based indicator of crop water status and irrigation response [49,52,53]. For instance, Zhang et al. [48] and Zhang et al. [49] demonstrated that reduced stomatal conductance and transpiration under water deficit conditions were associated with increased maize canopy temperature. Nevertheless, canopy temperature is influenced by factors beyond plant water status. Variables such as incoming solar radiation, air temperature, relative humidity, vapor pressure deficit, wind speed, and canopy architecture also affect the canopy energy balance and the resulting thermal response [53].

Further consideration of this interpretation is necessary for forage cactus, as *Opuntia* species exhibit CAM mechanism and possess physiological adaptations that promote water conservation under high evaporative demand and limited water availability. In CAM plants, stomatal activity and CO_2_ uptake primarily occur at night, altering the temporal relationship between gas exchange, transpiration, and environmental conditions compared to conventional C3 and C4 crops [54,55]. As a result, the association between daytime canopy temperature and instantaneous transpiration in forage cactus should be interpreted with caution. Gas-exchange responses also differ among genotypes; Silva et al. [56] reported significant variability in stomatal conductance, transpiration, and other gas-exchange traits among *Opuntia* and *Nopalea* cultivars. Martins et al. [57] further demonstrated that transpiration, stomatal conductance, and cladode temperature of forage cactus grown under semiarid conditions varied among clones and across seasons, underscoring the influence of both genotype and environmental conditions on plant thermal and physiological responses. Thus, the temperature predicted in the present study should be regarded as a thermal proxy reflecting crop–environment interactions, rather than as a direct indicator of plant water stress. Confirmation of water-stress status requires concurrent physiological or water-balance measurements, such as stomatal conductance, plant water potential, soil water content, evapotranspiration, or CWSI.

The observed differences among predictive approaches suggest that the relationship between UAV-derived spectral information and canopy temperature involves both complementary and potentially nonlinear spectral responses. The enhancement of multiparametric regressions with additional predictors demonstrates the value of incorporating multiple spectral features, which aligns with findings from previous UAV-based crop studies [50,58]. The higher mean performance of KNN (test R^2^ = 0.7811) results from its capacity to capture local relationships in the predictor space without requiring a predefined functional form. Nevertheless, this outcome should not be interpreted as evidence of the inherent superiority of KNN. Cruz-Grimaldo et al. [59] reported similar performances for RF (R^2^ = 0.80), KNN (R^2^ = 0.78), and gradient boosting (R^2^ = 0.79) in maize CWSI estimation, while Kapari et al. [58] achieved the highest performance with RF (R^2^ = 0.85) using multispectral and thermal UAV data. These findings indicate that algorithm performance is influenced by factors such as crop type, target variable, predictor set, sample structure, and environmental conditions, rather than by algorithm type alone.

The irrigation regime adopted in this experiment is important for interpreting model performance. Irrigation likely maintained plant water availability, thereby limiting the occurrence of the broad physiological gradients typically observed in controlled deficit-irrigation experiments. Consequently, a portion of the observed temperature variability may be attributed to differences in canopy development and immediate environmental factors, rather than pronounced differences in plant water status. This approach differs from studies that employ multiple irrigation levels to intentionally expand thermal and spectral responses across well-watered and water-stressed conditions. For instance, Wang et al. [50] integrated UAV multispectral and thermal data with direct measurements of stomatal conductance and soil water content under water-deficit treatments, showing that predictor sensitivity and model performance varied across crop developmental stages.

Semiarid atmospheric conditions introduce additional complexity to this relationship. Elevated solar radiation increases the radiative load on the canopy, while high air temperature and vapor pressure deficit intensify atmospheric evaporative demand. Relative humidity and wind speed further affect canopy energy exchange and, consequently, crop temperature [52,53]. These factors are particularly significant for forage cactus, as physiological responses such as transpiration, stomatal conductance, and cladode temperature can vary considerably across seasons and microclimatic conditions in semiarid environments [57]. As a result, similar spectral responses may correspond to different canopy temperatures under varying meteorological conditions. This environmental dependence is especially pertinent because the three UAV campaigns in this study were conducted under distinct environmental conditions. Therefore, the relationships identified should not be presumed stable across different irrigation regimes, seasons, or semiarid environments without independent validation.

Multispectral UAV imagery offers a complementary method for mapping spatial variability in forage-cactus canopy temperature when thermal imagery is unavailable. This approach can facilitate the identification of areas with contrasting thermal behavior and support targeted field assessments in irrigated semiarid environments [60]. Nevertheless, the proposed method should be regarded as a tool for canopy-temperature estimation, not as a replacement for thermal sensing or as a direct technique for diagnosing crop water stress. Its operational applicability requires independent validation across different cultivars, irrigation regimes, seasons, and environmental conditions.

Several limitations must be acknowledged when interpreting these results. First, the experiment was conducted at a single site and included three UAV acquisition campaigns within a specific irrigated forage-cactus production system. As a result, the dataset does not capture the full range of variability associated with different seasons, irrigation regimes, cultivars, soil conditions, or semiarid environments. Second, no independent external dataset was considered. Although 1500 randomized train/test partitions provide information on internal model stability, observations from the same experimental context do not constitute independent spatial or environmental validation. Therefore, the predictive performance reported here should be regarded as internal evidence of model potential rather than as confirmation of broad transferability.

Uncertainty may originate from both UAV image processing and reference-temperature measurements. UAV-derived spectral data are subject to influences such as illumination conditions, radiometric calibration, image mosaicking, georeferencing, and the presence of mixed pixels. In contrast, handheld thermal measurements can be affected by emissivity assumptions, viewing geometry, measurement distance, and environmental conditions during data acquisition. Previous UAV-based studies have shown that canopy structure and exposed soil can impact temperature retrieval. For instance, Zhang et al. [49] observed increased temperature residuals associated with variations in canopy cover and soil background. These sources of uncertainty highlight the necessity for standardized acquisition and preprocessing protocols when transferring predictive models across different sensors, sites, and environmental conditions [61]. It is recommended that future research incorporate multiple sites, seasons, cultivars, and irrigation regimes, along with independent external validation and simultaneous measurements of soil water content, stomatal conductance, plant water potential, evapotranspiration, and/or CWSI.

## 5. Conclusions

The potential of unmanned aerial vehicle (UAV)-derived multispectral vegetation indices and spectral bands for indirect estimation of canopy surface temperature in forage cactus irrigated with reused water in the Brazilian semiarid region was assessed. Vegetation indices demonstrated consistent and negative correlations with canopy temperature, suggesting that UAV-derived spectral data provide statistically significant information related to thermal variability in forage cactus under the studied conditions. Predictive performance of regression models improved as the number of predictor variables increased, with the seven-variable multiparametric model yielding the highest overall regression performance (validation R^2^ = 64.70%; Nash–Sutcliffe efficiency = 0.84). Among the machine learning algorithms tested, K-Nearest Neighbors achieved the highest predictive performance across 1500 randomized train/test partitions (mean test R^2^ = 78.11%), surpassing Random Forest, epsilon-support vector regression, and Extreme Gradient Boosting. When analysis was limited to RGB-derived indices, epsilon-support vector regression attained a R^2^ of 67.92%, demonstrating that relevant predictive information is also present in the visible spectral domain.

Collectively, the results suggest that UAV-derived spectral indices and bands provide meaningful information for the indirect estimation of forage cactus canopy temperature under the evaluated conditions. Nevertheless, these findings represent preliminary evidence of a promising proxy rather than a demonstration of sensor replacement. Independent validation across multiple seasons, irrigation regimes, cultivars, and environmental conditions is necessary before this approach can be considered a feasible operational alternative to direct thermal sensing. Thermal observations are essential for model calibration and independent validation prior to establishing the transferability of the proposed approach to different environmental and management conditions.

Furthermore, these findings should not be regarded as direct evidence of plant water status, as no direct physiological measurements of plant water relations were obtained. The research was limited to a single experimental site, three acquisition dates, one crop species, one UAV platform, and one thermal camera and lacked an independent external validation dataset. Future research should incorporate multiple sites, diverse sensor configurations, varied environmental conditions, and direct physiological measurements to more rigorously evaluate the robustness, transferability, and practical applicability of the proposed methodology.

## Figures and Tables

**Table 1:** Multispectral indices and bands used in the correlation analysis with biometric and temperature data.

Index	Name	Symbol/Equation	Reference
Bands			
R	Red	R	-
G	Green	G	-
B	Blue	B	-
RE	Red Edge	RE	-
NIR	Near Infrared	NIR	-
Multispectral			
NDVI	Normalized Difference Vegetation Index	$\frac{\mathrm{NIR}-R}{\mathrm{NIR}+R}$	Rouse et al. [27]
GNDVI	Green Normalized Difference Vegetation Index	$\frac{\mathrm{NIR}-G}{\mathrm{NIR}+G}$	Gitelson et al. [28]
NDRE	Normalized Difference Red Edge index	$\frac{\mathrm{NIR}-\mathrm{RE}}{\mathrm{NIR}+\mathrm{RE}}$	Tucker [29]
EVI	Enhanced Vegetation Index	$\frac{2.5\times (\mathrm{NIR}-R)}{\mathrm{NIR}+6\times R-7.5\times B}$	Huete et al. [30]
OSAVI	Optimized Soil Adjustment Index	$\frac{\mathrm{NIR}-R}{\mathrm{NIR}+R+0.16}$	Rondeaux et al. [31]
GCI	Green Chlorophyll Index	$\frac{\mathrm{NIR}}{G}-1$	Gitelson et al. [32]
NDVI_705_	Red-Edge Normalized Difference Vegetation Index	$\frac{\mathrm{RE}-R}{\mathrm{RE}+R}$	Evangelides and Nobajas [33]
RGB			
NDI	Normalized Difference Vegetation Index	$\frac{G-R}{G+R}$	Tucker [29]
IKAW	Kawashima index	$\frac{R-B}{R+B}$	Kawashima and Nakatani [34]
VARI	Visible Atmospherically Resistant Index	$\frac{G-R}{G+R-B}$	Gitelson et al. [35]

Where R = red band; G = green band; B = blue band; RE = red-edge band; NIR = near-infrared band.

**Table 2:** Hyperparameters and values evaluated for the optimization of the machine learning models.

Model	Tuning Parameters
Random Forest	n_estimators = 10, 50, 100, 200, 300, 500, 1000
Epsilon-Support Vector Regression	c = 0.1, 0.5, 1, 5, 10, 20, 50, 100
Extreme Gradient Boosting	n_estimators = 50, 100, 200, 300, 500
K-Nearest Neighbors	n_neighbors = 1 to 30

**Table 3:** Machine learning models and hyperparameters used.

All							
Random Forest		Epsilon-Support Vector Regression		XGBoost		K-Nearest Neighbors	
Hyperparameter	Value	Hyperparameter	Value	Hyperparameter	Value	Hyperparameter	Value
N_estimators	1000	Kernel	Rbf	N_estimators	300	n_neighbors	8
Random_state	0	C	5	Random_state	0		
				Objective	reg:squarederror		
				Learning_rate	0.01		
**RGB only**							
**Hyperparameter**	**Value**	**Hyperparameter**	**Value**	**Hyperparameter**	**Value**	**Hyperparameter**	**Value**
N_estimators	1000	Kernel	Rbf	N_estimators	200	n_neighbors	8
Random_state	0	C	1	Random_state	0		
				Objective	reg:squarederror		
				Learning_rate	0.01		

**Table 4:** Descriptive statistics for the temperature and indices.

	Mean	Median	Maximum	Minimum	Standard Deviation	CV	1º Quartile	3º Quartile
Tm	39.372	38.125	46.150	31.850	3.795	0.096	36.375	43.200
NDVI	0.596	0.614	0.840	0.287	0.126	0.212	0.508	0.695
GNDVI	0.457	0.523	0.800	−0.025	0.200	0.438	0.290	0.613
NDRE	0.134	0.141	0.347	−0.078	0.081	0.603	0.083	0.192
EVI	1.343	2.036	6.969	−5.269	2.095	1.560	−0.759	2.720
OSAVI	0.596	0.614	0.840	0.287	0.126	0.212	0.508	0.695
NDVI705	0.511	0.534	0.719	0.170	0.107	0.209	0.441	0.594
GCI	2.650	2.272	10.476	−0.048	2.084	0.786	0.815	3.927
NDI	0.189	0.194	0.390	−0.028	0.099	0.523	0.121	0.259
IKAW	−0.052	−0.009	0.212	−0.336	0.163	3.158	−0.230	0.086
VARI	0.400	0.308	1.016	−0.042	0.277	0.692	0.218	0.634
R	6336.578	4542.000	17,211.000	736.000	4364.490	0.689	2977.500	10,048.750
NIR	22,488.178	21,266.500	44,680.000	5053.000	9064.093	0.403	16,610.000	28,654.000
RE	18,171.289	15,397.500	41,049.000	3104.000	9633.516	0.530	11,600.250	24,597.500
G	10,391.033	6279.000	31,526.000	963.000	8737.808	0.841	3905.500	18,010.000
B	9004.811	4256.000	27,563.000	886.000	8531.024	0.947	2805.000	17,314.750

Where Tm: temperature (°C), CV: coefficient of variation.

**Table 5:** ANOVA statistical analysis.

ANOVA							
**20/01/2026**							
S.V.	D.F.	S.S.	M.S.	F		f 5%	f 1%
Block	4	17,655.21	4413.80	8.44	**	2.8	4.26
Treatment	2	1.91	0.96	0.00	NS	3.42	5.66
Residual	23	12,033.27	523.19				
Total	29	29,690.39					
**25/02/2026**							
Block	4	26,032.31	6508.08	8.45	**	2.8	4.26
Treatment	2	0.45	0.22	0.00	NS	3.42	5.66
Residual	23	17,706.67	769.86				
Total	29	43,739.42					
**25/05/2026**							
Block	4	18,772.32	4693.08	8.13	**	2.8	4.26
Treatment	2	3.12	1.56	0.00	NS	3.42	5.66
Residual	23	13,284.76	577.60				
Total	29	32,060.21					

S.V.: source of variation; D.F.: degrees of freedom; S.S.: sum of squares; M.S.: mean square. ** *p* < 0.01.

**Table 6:** Regression models for canopy temperature prediction using multispectral bands and vegetation indices, together with their performance metrics.

Model	Equation	R^2^ Calibration	R^2^ Validation	Adjusted R^2^	Nash–Sutcliffe	PBIAS
Simple	*Tm* = 0.713 × *B* + 0.305	75.81%	50.04%	47.41%	0.7805	0.2624%
Quadratic	*Tm* = 1.08 − 2.307 × *EVI* + 1.719 × *EVI*^2^	79.83%	64.13%	62.24%	0.8304	1.5564%
Cubic	*Tm* = −2.571 × *B*^3^ + 3.196 × *B*^2^ − 0.181 × *B* + 0.341	80.91%	57.32%	55.08%	0.8023	0.1849%
MP 2	*Tm* = 0.531 − 0.407 × *EVI* + 0.495 × *B*	79.86%	58.75%	54.16%	0.8185	0.7252%
MP 3	*Tm* = 0.292 + 0.347 × *NDRE* − 0.379 × *EVI* + 0.677 × *B*	81.92%	61.53%	55.09%	0.8316	0.4999%
MP 4	*Tm* = 0.460 + 0.304 × *NDRE* − 0.38 × *EVI* − 0.181 × *IKAW* + 0.51 × *B*	82.88%	60.69%	51.06%	0.8276	0.2565%
MP 5	*Tm* = 0.618 − 0.365 × *GNDVI* + 0.448 × *NDRE* − 0.326 × *EVI* − 0.168 × *IKAW* + 0.365 × *B*	83.66%	61.29%	48.43%	0.8262	0.3312%
MP 6	*Tm* = 0.696 − 0.538 × *GNDVI* + 0.423 × *NDRE* − 0.312 × *EVI* + 0.188 × *GCI* − 0.177 × *IKAW* + 0.320 × *B*	84.02%	63.03%	47.14%	0.8323	0.2683%
MP 7	*Tm* = 0.863 − 0.602 × *GNDVI* + 0.439 × *NDRE* − 0.344 × *EVI* + 0.253 × *GCI* − 0.302 × *IKAW* − 0.179 × *VARI* + 0.313 × *B*	84.46%	64.70%	45.29%	0.8408	0.2544%

**Table 7:** Regression models and performance evaluation metrics for RGB indices.

Model	Equation	R^2^ Calibration	R^2^ Validation	Adjusted R^2^	Nash–Sutcliffe	PBIAS
Simple	*Tm* = −0.727 × *IKAW* + 0.912	67.37%	42.31%	39.28%	0.6197	−1.3309%
Quadratic	*Tm* = 0.995 − 1.35 × *IKAW* + 0.66 × *IKAW*^2^	70.38%	52.60%	50.11%	0.7869	−0.7813%
Cubic	*Tm* = 3.433 × *IKAW*^3^ − 4.6 × *IKAW*^2^ + 0.805 × *IKAW* + 0.835	75.93%	55.70%	53.37%	0.8015	−0.1809%
MP 2	*Tm* = 0.763 − 0.586 × *IKAW* + 0.178 × *VARI*	67.90%	42.86%	39.85%	0.7354	−1.2603%
MP 3	*Tm* = 0.735 − 0.117 × *NDI* − 0.535 × *IKAW* + 0.326 × *VARI*	68.04%	44.70%	41.79%	0.7461	−1.1983%

**Table 8:** Performance metrics for the machine learning models.

Algorithm	MAE		RMSE		R^2^	
	Mean	Sd	Mean	Sd	Mean	Sd
All bands and indices						
RF	1.4211	0.2066	1.7882	0.2470	0.7583	0.0832
XGBoost	1.6287	0.2727	2.0574	0.3623	0.6764	0.1357
SVR	1.4384	0.2059	1.7998	0.2374	0.7561	0.0800
KNN	1.3187	0.2129	1.6996	0.2526	0.7811	0.0796
RGB only						
RF	1.596	0.2498	2.1248	0.3426	0.6578	0.1251
XGBoost	1.7048	0.2752	2.2392	0.3710	0.6212	0.1410
SVR	1.6350	0.2435	2.0702	0.3209	0.6792	0.1036
KNN	1.5100	0.2498	2.0083	0.3635	0.6942	0.1158

## Data Availability

The data presented in this study are available upon request from the corresponding author.

## Abbreviations

The following abbreviations are used in this manuscript:

B	Blue
CV	Coefficient of variation
G	Green
KNN	K-Nearest Neighbors
MAE	Mean Absolute Error
MAV	Mimoso Alluvial Valley
NIR	Near Infrared
PE	Pernambuco
R	Red
R^2^	Coefficient of determination
RE	Red Edge
RF	Random Forest
RMSE	Root Mean Squared Error
SVR	Support Vector Regression
UAVs	Unmanned aerial vehicles
XGB	Extreme Gradient Boosting
